# Hypoparathyroidism and late-onset hypogonadism in an adult male with familial 22q11.2 deletion syndrome: a case report with 3-year follow-up and review of the literature

**DOI:** 10.1186/s12902-022-01150-z

**Published:** 2022-11-12

**Authors:** Xuelian Chen, Lichuan Yang, Jianwei Li, Huiwen Tan

**Affiliations:** 1 Department of Endocrinology and Metabolism, West China Hospital, Sichuan University, Chengdu, 610041 Sichuan China; 2 Department of Nephrology, West China Hospital of Sichuan University, Chengdu, 610041 Sichuan China

**Keywords:** 22q11.2DS, DiGeorge syndrome, Hypogonadism, Hypocalcemia, Hypoparathyroidism, Metabolic syndrome, Chronic kidney disease, Case report

## Abstract

**Background:**

22q11.2 deletion syndrome (DiGeorge syndrome) is associated with multiple organ dysfunctions such as cardiac defects, immunodeficiency, and hypoplasia of parathyroid glands. Moreover, the phenotype of 22q11.2 DS has clinical variability and heterogeneity.

**Case presentation:**

In this report, we present the case of a 35-year-old patient with a past medical history that included recurrent infections, mild learning difficulties in childhood, pediatric obesity, and cataract. He was admitted to the endocrinology department for the management of hypogonadism and hypocalcemia. During the 3-year follow-up, the patient gradually developed primary hypoparathyroidism, hypogonadism, chronic renal failure, and heart failure, and his medical condition deteriorated. Meanwhile, in order to improve clinicians’ awareness of the endocrine manifestations of adult 22q11.2 DS and reduce missed diagnoses, we reviewed 28 case reports of adult 22q11.2 DS to analyze the clinical characteristics.

**Discussion:**

Here, we report the case of a young man diagnosed with 22q11.2 DS presented a rare combination of multiple endocrine disorders. This is the first time that a patient with 22q11.2DS had late-onset hypogonadism caused by primary testicular failure combined with decreased pituitary gonadotropin reserve in a patient with 22q11.2DS.

## Background

22q11.2 deletion syndrome (22q11.2DS), associated with multi-organ dysfunction including cardiac defects, immunodeficiency, and hypoplasia of parathyroid glands, is the most common chromosome microdeletion syndrome. Clinical epidemiological studies revealed that the prevalence of the syndrome is estimated to be 1 in 3,000 to 6,000 live births, and this chromosomal microdeletion disorder could be present at any age. The majority of newly discovered patients with 22q11.2DS (90–95%) have spontaneous mutations [1]. Familial occurrence is the most common indication for adult 22q11.2DS to be referred to the genetic clinic [2]. The phenotype of patients with 22q11.2DS has clinical variability and heterogeneity. DiGeorge syndrome is defined as a combination of immune abnormalities, hypoparathyroidism, and complex heart disease [3–5]. Currently, there is limited understanding of 22q11.2DS and its broad phenotypic spectrum. Many adults with 22q11.2DS have not yet been diagnosed, and the life expectancy of adults with 22q11.2DS is shortened, and the risk of sudden death increases [6–8]. 22q11.2DS is the leading cause of congenital hypoparathyroidism, and affected patients show a series of autoimmune characteristics. The syndrome is evident in early childhood and is rarely diagnosed in adulthood [9]. There is no previous report of hypogonadism in patients with 22q11.2DS. Here, we present the first case of a patient with 22q11.2DS and hypogonadism.

## Case presentation

A 35-year-old male was admitted to the department of endocrinology and metabolism with diarrhea for 1 week. The patient had diarrhea 5—6 times per day, along with nausea and vomiting, decreased urine output, edema, and lower limb fatigue. Laboratory examinations suggested hypocalcemia and hyperphosphatemia, chronic renal failure, and heart failure. After admission to the hospital for calcium supplementation and diuresis, re-assessment showed that the total serum calcium concentration increased to 2.35 mmol/L (normal range: 2.11- 2.52 mmol/L), and the N-terminal pro-B-type natriuretic peptide (NT-proBNP) concentration fell to 5304 ng/mL (normal range: 0—88 ng/mL).

Notably, the patient’s two kids were diagnosed with congenital heart disease successively, thus the patient and his children were diagnosed as 22q11.2DS by genetic testing in 2013. Furthermore, the patient was repeatedly hospitalized with Type 2 diabetes (T2DM) and hypogonadism. In 2015, the patient at the age of 28 years old had sexual dysfunction including erectile dysfunction, ejaculatory dysfunction, and decreased libido. In 2016, the patient’s physical examination revealed borderline elevated blood prolactin (PRL: 21.72 ng/ml) and normal testosterone levels (T: 2.70 ng/ml) for which bromocriptine was administered for 3 months at other hospital. He was diagnosed as having a pituitary adenoma, because enhanced MRI of the pituitary revealed pituitary space-occupying lesions. In 2017, the testosterone test showed a decrease, and he was given testosterone undecanoate soft capsules for one month. Testosterone and PRL levels were not monitored while the patients were taking bromocriptine and testosterone undecanoate soft capsules. The patient ejaculated after 1 sexual intercourse after 10 days of continuous human chorionic gonadotropin (HCG) and urinary gonadotropin injections in 2018. Until the present admission, the patient’s hypogonadism had not improved.

His medical history included recurrent infections, mild learning difficulties in childhood, pediatric obesity, and cataracts. He denied the history of exposure to toxicants. His father had diabetes. The mother lived in the worker dormitory of a factory producing plastic during pregnancy. The patient’s first kid with recurrent chronic infections was diagnosed as having congenital heart disease and 22q11.2 microdeletion syndrome. The patient’s wife had an abortion after the youngest kid was found to have congenital heart disease during a maternity checkup.

### Physical examination

Vital signs T 36.4℃, P 89 bpm, R 20 bpm, BP 109/80 mmHg, height 165 cm, weight 80 kg, body mass index (BMI) 29.38 kg/m^2^, waist 95 cm, hip 110 cm. Waist to hip ratio 0.86.

Dysmorphic facies moon-face, short eyelid cleft, wide eye distance, low nose bridge, different shapes and sizes of ears, left helix curved to the ventral side.

### Physical findings

Sparse eyebrows, beard, pubic hair, and axillary hair, bilateral breast development, bulging abdomen, white stripes on both sides of the abdomen wall, the widest is about 2–3 mm. There are 10 cm and 15 cm old scars on the left upper abdomen. Mild depression and edema in the front tibia of both lower limbs. The penis is about 3.5 cm long and 2 cm in diameter, the left testis is 2.5 cm long, the right testis is about 2.3 cm long, and there is an old scar about 2 cm long on the right side of the scrotum. The Chvostek’s sign and the Trousseau sign were negative.

### Genetic analysis

**Fig. 1:**
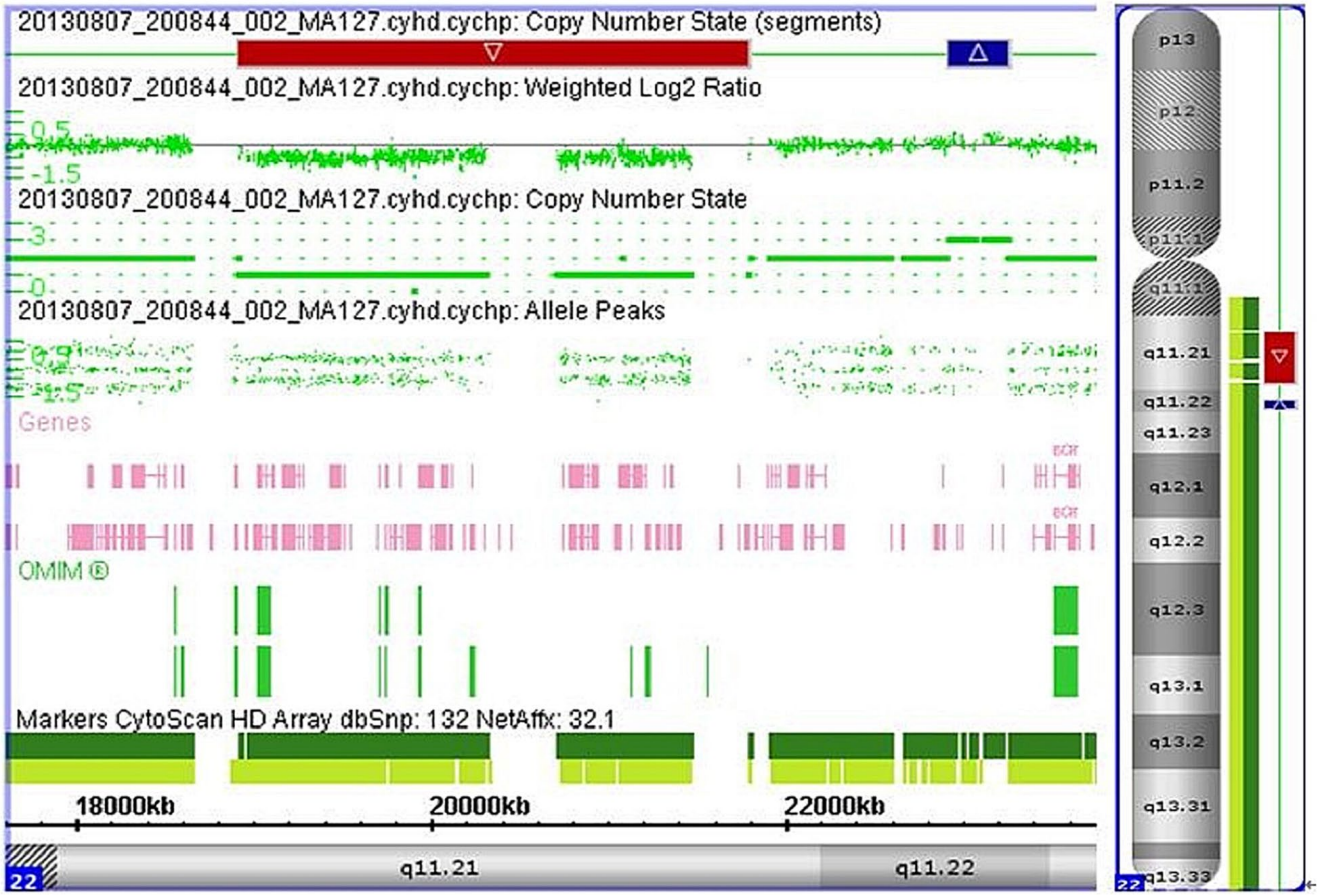
Genetic analysis. Note: Red markers represented gene deletions and blue markers represented gene duplications

The patient’s genetic test showed chr22:18,916,842–21,800,797 (hg19) with a 2,884-Kb chr22q11.21 deletion, encompassing 41 Online Mendelian Inheritance in Man (OMIM) genes (Fig. 1).

The genetic test of the patient’s eldest son showed an arr [hg19] 22q11.21 (18,648,855–21,800,471) × 1 with a 3152-Kb chr22q11.21 deletion encompassing 46 Online Mendelian Inheritance in Man (OMIM) genes, including TBX1 (OMIM: 602,054) and encompassing 95 genes in the Decipher database.

### Laboratory examination

Of the three hospitalizations for this patient from 2017 to 2021, the patient’s labs have evolved (Tables 1 and 2). Peripheral blood lymphocyte culture chromosome karyotype analysis revealed a karyotype of 46, XY in 2017. Upper and lower limbs showed peripheral neurogenic damage (sensory fiber damage). Abdominal color Doppler ultrasound showed a fatty liver. Electroencephalography (EEG) in 2021 revealed a non-specific EEG finding.

**Table 1:** Laboratory data in this patient with 22q11.2 deletion syndrome

Items	Normal range	2017	2019	2021
RBC (10^9/L)	4.3–5.8	4.82	4.60	**2.91**
HB (g/L)	130–175	145	**113**	**86**
PLT (10^9/L)	100–300	83	138	273
CHOL (mmol/L)	2.8–5.7	**8.87**	**9.61**	5.21
TG (mmol/L)	0.29–1.38	**15.80**	**15.20**	**4.0**
HDL-C (mmol/L)	> 0.9	**0.72**	**0.7**	**0.61**
SCr (umol/L)	68–108	80.0	**147**	**594**
eGFR (ml/min/1.73m2)	56–122	113.45	**60.51**	**9.77**
Ca (mmol/L)	2.11–2.52	2.29	2.19	**1.03**
P (mmol/L)	0.81–1.45	1.37	1.30	**2.46**
ALP (IU/L)	51–160	130	70	109
PTH (pmol/L)	1.60–6.90	2.23	3.66	3.10
25-OH-VD (noml/L)	47.7–144	-	**20.09**	**11.8**
β-CTX (ug/L)	9.06–76.24	-	-	31.6
B-ALP (ng/ml)	11.4–24.6	-	17.04	15.15
tP1NP (pg/L)	0.300–0.584	-	**0.313**	0.437
U-PRO (g/L)	nagative	** + + **	** + **	** + + **
ALB/Cr (mg/g)	< 30	**101.5**	**186.6**	**337.3**
24 h-u-pro (g/24 h)	< 0.15	-	**1.44**	**9.36**
T cell count (cell/ul)	CD3 941–2226 CD4 471–1220 CD8 303–1003	-	CD3 1102 CD4 650 CD8 342	CD3 1545 CD4 1058 CD8 398
CD4/CD8	0.79–2.31	-	**2.41**	**2.66**
ANA	negative	-	** + **	** + **
Myo (ng/ml)	< 72	-	< 21	**243**
TPN-T (ng/L)	0–14	-	13.4	**49.8**
NT-proBNP (ng/mL)	0–88	-	75	**6099**
T (ng/ml)	2.49–8.36	**1.90**	**2.20**	**1.54**
Biologically active testosterone	-	62.93%	65.83%	53.54%
SHBG (nmol/L)	18.3–54.1	**13.35**	**12.37**	27.5
LH (mIU/L)	1.7–8.6	6.2	**10.8**	**12.9**
FSH, mIU/L	1.5–12.4	5.1	5.7	7.1
PRL, ng/ml	4.6–21.4	20.06	**22.48**	**32.6**
HTG, ug/L	1.4–78	**0.26**	-	**0.31**
GnRH test	Peak LH: 4—10 times more than basic LH Peak FSH: 0.5–2 times more than basic FSH	Basic LH: 9.5 Peak LH: 70.8 Time to peak LH: 30 min after the Intravenous injection of GnRH Basic FSH: 6.3 Peak FSH: 13.8 Time to peak FSH: 60 min after the Intravenous injection GnRH	Basic LH: 14.5 Peak LH: 83.2 Time to peak LH: 45 min after the Intravenous injection of GnRH Basic FSH: 7.49 Peak FSH: 16.2 Time to peak LH: 90 min after the Intravenous injection of GnRH	-
HCG stimulation test	Peak T: 2 times more than basic T		Basic T: 2.80 Peak T: 5.93 Basic FT: 3.15% Lowest FT: 2.17%	

*Abbreviations*: *RBC* Red blood cell, *HB* Hemoglobin, *PLT* Platelet, *CHOL* Cholesterol, *TG* Triglyceride, *HDL-C* High-density liptein cholesterol, *eGFR* Estimated glomerular filtration rate, *Scr* Serum creatinine, *Ca* Calcium, *P* Phosphate, *ALP* Alkaline phosphatase, *PTH* Parathyroid hormone, *25-OH-VD* 25-(OH) vitamin D, *beta-CTX* C-terminal telopeptide of beta-I collagen, *P1NP* Procollagen type 1 N-terminal propeptide, *B-ALP* Bone-specific alkaline phosphatase, *U-PRO* Urine protein, *ALB/C*r Albumin/creatinine ratio, *24 h-u-pro* 24 h urine protein quantitation, *ANA* Antinuclear antibody, *IgA* Immunoglobulin A, *Myo* Myoglobin, *TPN-T* Troponin T, *NT-proBNP* N-terminal pro-B-type natriuretic peptide, *T* Testosterone, *FT* Free testosterone, *SHBG* Sex hormone-binding globulin, *LH* Luteinizing hormone, *FSH* Follicle-stimulating hormone, *PRL* prolactin, *HTG* Human thyroglobulin, *GnRH* Gonadotropin-Releasing Hormone, *HCG* Human chorionic gonadotropin

- means that the laboratory data were not obtained

**Table 2:** Imaging examination in this patient with 22q11.2 deletion syndrome

	Normal	2017	2019	2021
Enhanced MRI of pituitary	normal	Low signal nodule 0.4 cm in diameter	Low signal nodule 0.5 cm in diameter	-
Echocardiography	LA 28–32 mm LV 47 mm EF 61%	-	LA 36 mm LV 47 mm EF 61%	LA 39 mm LV 55 mm EF58%
Holter ECG of HRV	normal	moderate reduction	severe reduction	severe reduction
OSAHS	-	moderate	Severe	Severe
Thyroid ultrasound	normal	Right thyroid nodule (10 × 5 × 7 mm) nodular goiter?	Right thyroid nodule (13 × 8 × 10 mm nodular goiter?	-
Testicular Sonographic Color Doppler ultrasound	Bilateral testicles size 40 × 30 × 30 mm volume 15–20 ml	left testis size 20 × 15 × 33 mm volume 7 ml right testis size 23 × 16 × 31 mm volume 8 ml	left testis size 20 × 14 × 31 mm volume 6.1 ml right testis size 23 × 16 × 30 mm volume 7.8 ml	-

*Abbreviations*: *ECG* Electrocardiogram, *MRI* Magnetic resonance imaging, *HRV* Heart rate variability, *OSAHS* Obstructive sleep apnea hypopnea syndrome, *LV* Left ventricular, *LA* Left atrium, *LVEF* Left ventricular ejection fraction

- means that the laboratory data were not obtained

## Discussion and conclusions

We reported the case of a young man diagnosed with 22q11.2 DS presented a rare combination of multiple endocrine disorders. The patient did not have the typical symptoms of the 22q11.2DS, only mild facial dysmorphism, learning difficulties, and recurrent infections as a child. He had T2DM, obesity, hypertension, dyslipidemia, hypocalcemia, primary hypoparathyroidism, and late-onset hypogonadism (LOH). Hypogonadism has not been previously reported in an adult with 22q11.2DS.

In order to improve clinicians’ awareness of the endocrine manifestations of adult 22q11.2 DS, we reviewed the available articles in the PubMed database for the past 20 years, found 2753 related articles, and finally screened out 28 case reports of adult 22q11.2 DS. The inclusion criteria were as follows: i) Patients diagnosed with 22q11.2 DS for the first time at age > 18 years. ii) The clinical events of 22q11.2 DS were mainly endocrine disease manifestations. The characteristics of 28 cases are summarized in Table 3.

**Table 3:** Summary of 28 cases of DiGeorge syndrome in adults with clinical manifestations

**Study**	**Time**	**country**	**Gender**	**Age**	**Endocrine manifestations**	**Other manifestations**	**Familial** **22q11.2DS**	**Treatment**	**Fellow-up**
P1 [10]	2005	England	Female	24	Seizure (hypocalcemia) Hypoparathyroidism	Learning difficulties Recurrent infections Electrocardiogram revealed a prolonged QT interval	Yes, She and her baby	Vitamin D	No
P2 [10]	2005	England	Female	52	Hypocalcemia Hypoparathyroidism	Facial dysmorphism Patent ductus arteriosus Cervical lymphadenopathy	No	Not mentioned	No
P3 [11]	2006	Italy	Male	19	Seizure (hypocalcemia) Hypoparathyroidism Vitamin D deficiency Parathyroid and thymus Hypoplasia Metabolic syndrome (insulin resistance, obesity, hypertriglyceridemia)	Facial dysmorphism Intellectual disability Multiple immune-related skin lesions Vasculitis Thrombocytopenia Antiphospholipid syndrome Hyperhomocysteinemia Hearing loss	No	Not mentioned	No
P4 [12]	2007	America	Female	32	Hypocalcemia	Learning difficulties Atrial septal defect Ventricular septal defect Bacterial endocarditis Velopharyngeal incompetence	Yes, She and her son	Calcium Vitamin D	No
P5 [13]	2007	America	Male	40	Seizure (hypocalcemia) Hypoparathyroidism	Basal ganglia calcification ECG revealed a prolonged QT interval	No	Calcium citrate Calcitriol	No
P6 [14]	2008	England	Female	29	Spasms (hypocalcemia)	Facial dysmorphism Learning difficulties Language delay Velopharyngeal incompetence	No	Calcium Vitamin D	No
P7 [15]	2004	America	Male	32	Seizure (hypocalcemia) Hypoparathyroidism Short stature	Facial dysmorphism Learning difficulties Hypernasal speech	No	Calcium citrate Valproic acid	No
P8 [16]	2010	Italy	Male	71	Hypocalcemia Hypoparathyroidism	Facial dysmorphism Intellectual disability Anxious-depressive syndrome Parkin-sonism syndrome Basal ganglia calcification Cerebral dysrhythmia	No	Calcium citrate	No
P9 [17]	2011	Australia	Male	40	Seizure (hypocalcemia) Hypoparathyroidism Vitamin D deficiency Developmental delay Osteoporosis	Facial dysmorphism Intellectual disability Childhood asthma Basal ganglion calcification	No	Calcium carbonate Calcitriol	No
P10 [18]	2012	China	Female	32	Chest pain(hypocalcemia) Seizure (hypocalcemia) Hypoparathyroidism	Facial dysmorphism Intellectual disability Learning difficulties Tetralogy of Fallot Hypernasal speech Electrocardiogram revealed a prolonged QT interval	No	Calcium gluconate	Yes, 9 months
P11 [19]	2013	Thailand	Male	26	Carpopedal spasm (hypocalcemia) Hypoparathyroidism Short stature Obesity	Facial dysmorphism Intellectual disability Hearing loss Basal ganglion calcification	No	Calcium carbonate Alfacalcidiol	Yes
P12 [20]	2013	Japan	Female	36	Hypocalcemia Hypoparathyroidism Hashimoto’s thyroiditis T2DM (diagnosed at 20 years old) Obesity	Facial dysmorphism Learning difficulties Tetralogy of Fallot Patellar dislocation Hearing loss	No	Calcium lactate Calcitriol Thyroid hormone replacement Hypoglycemia agent	No
P13 [21]	2013	Portugal	Male	34	Loss of consciousness (hypocalcemia) Hypoparathyroidism Enlarged thyroid gland Follicular adenomata Delayed growth	Facial dysmorphism Learning difficulties Right-sided aortic arch Recurrent infections Multiple operations (testicular torsion, acute appendicitis, congenital epigastric hernia, supraumbilical hernia, accessory digit)	No	Calcium Alpha-calcidol Antiepileptic drugs	Yes
P14 [22]	2015	America	Male	29	Hypocalcemia Acute Hypercalcemia (Excessive intake)	Tetralogy of Fallot Esophageal stricture Milk-alkali syndrome (MAS) hypercalcemia-induced pancreatitis	No	calcitonin	No
P15 [23]	2015	Japan	Male	39	Seizure (hypocalcemia) Hypoparathyroidism	Facial dysmorphism Learning difficulties Delayed speech Cerebellar development disorder Anxiety disorder Otosclerosis Cataract ECG revealed a prolonged QT interval	No	Calcium Calcitoriol	No
P16 [24]	2015	Japan	Male	40	Seizure (hypocalcemia) Hypoparathyroidism Obesity Short structure	Facial dysmorphism Intellectual disability Psychosis Tetralogy of Fallot Velopharyngeal incompetence Hyperprolinemia Basal ganglion calcification ECG revealed a complete right bundle branch block	No	Antipsychotic drugs Antiepileptic drugs alfacalcidol	No
P17 [25]	2016	Japan	Male	49	Seizure (hypocalcemia) Hypoparathyroidism Vitamin D deficiency	Facial dysmorphism Intellectual disability Psychosis Velopharyngeal incompetence Cataract	No	Vitamin D Calcium gluconate	Yes, 1 year
P18 [26]	2017	Turkey	Male	35	Azoospermia	Facial dysmorphism Intellectual disability hypernasal speech	No	Not mentioned	No
P19 [27]	2017	Japan	Male	54	Hypocalcemia Hypoparathyroidism	Facial dysmorphism cardiac anomalies (high-positioned RAA, ALSA, KD, and ASLBV) Lymphocytopenia	No	Not mentioned	No
P20 [28]	2018	Portugal	Male	58	Spasms, dyspnea(hypocalcemia) Hypoparathyroidism Sparse hair Micropenis (Fertility function not mentioned)	Facial dysmorphism Intellectual disability Psychosis Velopharyngeal incompetence Hearing loss Cataract ECG revealed a prolonged QT interval	Suspected	calcium gluconate, calcium carbonate, cholecalciferol	Yes, 8 months
P21 [29]	2018	Spain	Male	57	Perioral and finger numbness(hypocalcemia) Hypoparathyroidism T2DM Obesity	Learning difficulties Intellectual disability Behavioral disturbance Recurrent infections Renal carcinoma Lymphocytopenia Hypertension Hypercholesterolemia	No	intravenous calcium, oral magnesium oral calcitriol	No
P22 [30]	2018	Portugal	Male	30	generalised choreiform dyskinesias (hypocalcemia) Vitamin D deficiency	Facial dysmorphism Learning difficulties Psychosis Parkinson’s disease	No	calcium carbonate calcitriol	Yes, 1 year
P23 [31]	2018	China	Female	62	Hypocalcemia, Hypoparathyroidism Short stature Obesity	Facial dysmorphism Intellectual disability Schizophrenia Lymphocytopenia Macrothrombocytopenia Hearing loss Impaired renal function Hyperphosphatemia	No	calcium calcitriol	No
P24 [32]	2020	Japan	Female	44	Seizure (hypocalcemia) Hypoparathyroidism OYL, ventriculomegaly of the brain and lower thoracic spinal stenosis (secondary to hypoparathyroidism)	Facial dysmorphism Ventricular septum defect (cardiac VSD) Schizophrenia Deafness Cataract	No	Alfacalcidol calcium lactate	No
P25 [33]	2020	Korea	Male	36	Seizure (hypocalcemia) Hypoparathyroidism	Facial dysmorphism Impaired renal function Hyperphosphatemia ECG revealed a prolonged QT interval		calcium carbonate, calcitriol, valproate,	No
P26 [34]	2021	India	Male	40	Seizure (hypocalcemia) Hypoparathyroidism Short stature	Facial dysmorphism Learning difficulties Cataract Hyperphosphatemia Basal ganglion calcification ECG revealed a prolonged QT interval	No	calcium gluconate calcium vitamin D analogues	Yes
P27 [35]	2021	Germany	Female	44	Recurrent episodes of syncope (hypocalcemia) Hypoparathyroidism	ECG revealed a prolonged QT interval	No	vitamin D calcium Levetiracetam	No
P28 [36]	2021	Netherlands	Male	56	Unconsciousness (hypocalcemia) Hypoparathyroidism Vitamin D deficiency	Intellectual disability Schizophrenia Velopharyngeal incompetence Psoriasis Cataract Renal insufficiency Hyperphosphatemia	No	calcium gluconate calcium carbonate alfacalcidol	

Among the 28 published cases we reviewed, 7 patients were diagnosed for the first time at age > 50 years (7/28). The maximum age at the time of the first diagnosis of 22q11.2 DS was 71 years old. The main manifestations of the endocrine system are as follows: seizures or convulsions caused by hypocalcemia or primary hypoparathyroidism (17/28), short stature or developmental delay (6/28), obesity (6/28), T2DM (2/28), Hashimoto’s thyroiditis (1/28), thyroid cancer (1/28), micropenis (1/28), azoospermia (1/28), metabolic syndrome (1/27), insulin resistance (1/28). Although 20 of the 28 patients had facial dysmorphism, 10 patients had varying degrees of intellectual disability, and 10 patients had psychosis, their diagnoses were still delayed. 22q11.2DS presents multisystem features in which the endocrine system is characterized by hypocalcemia or hypoparathyroidism (> 60%), adult obesity (35%), and T2DM is a rare feature of 22q11.2DS [37]. Adults with 22q11.2DS are more likely to develop T2DM and obesity at a younger age [38, 39].

Phenotypes of 22q11.2DS have extensive heterogeneity and variability. It usually comes in the form of the triad of DiGeorge syndrome, which includes immunodeficiency, congenital heart defects, and hypocalcemia caused by hypoparathyroidism [1, 3]. Hypoparathyroidism is a rare endocrine disease characterized by hypocalcemia. Hypoparathyroidism caused by DiGeorge syndrome accounts for 60% of hypoparathyroidism in children [40]. Hypocalcemia in patients with 22q11.2DS can occur at any age, usually in the neonatal period, and rarely in adults [9]. Because of the 22q11.2DS phenotypic variability, mild symptoms may be missed clinically [37]. In the review of 28 cases, we found that more than half of the patients presented with symptomatic hypocalcemia, which manifested as limb twitching, seizures, and sudden loss of consciousness. They were also seen in different departments as a result, including neurology, psychiatry, and endocrinology, and most of them received calcium supplementation and were eventually diagnosed with 22q11.2DS. Giving 10% calcium gluconate and calcium supplementation, vitamin D supplementation, and vitamin analogues are all important ways to treat hypocalcemia or hypoparathyroidism [40].

The young patient that we reported had sexual dysfunction, bilateral testicular atrophy, with testicular volume of 6–8 mL at the age of 28. Human chorionic gonadotropin (HCG) stimulation test was performed to assess the testicular function. After intramuscular administration of HCG 2000 units for three days, testosterone (T) levels were elevated less than two times, and free testosterone (FT) levels were reduced. These results all indicated that the patient had LOH caused by primary testicular failure.

It is worth exploring that LH and FSH levels were not significantly elevated as would be expected in primary hypogonadism. Studies indicated that obesity, T2DM, metabolic syndrome, chronic kidney disease, sleep disorders, and hyperprolactinemia were common causes of secondary hypogonadism [41–43]. The prevalence of hypogonadism in overweight men was high, especially in patients with type 2 diabetes combined with obesity [42]. Multiple conditions including hyperprolactinemia, obesity, and T2DM that can result in secondary hypogonadism were present in the patient. Prolactin was found to be only transiently and borderline elevated in 2015 at other hospital. Prolactin levels tested at our hospital in 2017 were normal and the patient developed proteinuria, suggesting chronic kidney disease (CKD) stage 2. In the following years, as renal failure progressed, the patient experienced borderline elevated prolactin levels. Studies have shown that pharmacological renal insufficiency may lead to elevated prolactin. In approximately one-third of patients with renal disease, hyperprolactinemia occurs due to reduced clearance and increased hormone production [44]. Therefore, the borderline elevated prolactin in this patient was associated with renal failure.

Moreover, the patient’s last GnRH stimulation test suggested a peak FSH increase of approximately 4.73-fold, which was relatively low within normal limits. This revealed that the pituitary gonadotropin reserve decreased as the disease advanced. In summary, the patient had primary hypogonadism combined with decreased reserve function of the hypothalamo-pituitary–gonadal axis.

In the cases we reviewed (Table 3), one patient had a micropenis, but the patient’s marriage and childbirth history were not mentioned in the case report, and it was not clear whether the patient’s fertility was affected. An adult patient with 22q11.2DS reported in Turkey had azoospermia, leading to infertility. This suggests that male infertility may be an under-recognized phenotype associated with 22q11.2 deletion syndrome.

Additionally, synaptosomal-associated protein 29 (SNAP29) has been found to be absent in 90% of 22q11.2DS patients. Snap29-/- mutant male mice have a significantly reduced testis/body ratio, abnormal spermatogenesis, and no live births were found in the offspring. This indicated that SNAP29 is essential for male fertility and spermatogenesis [45]. Other study also indicated that patients with typical LCR22 A-D deletions may present with cryptorchidism [46]. Furthermore, fetal loss or infant death is a rare symptom in patients with 22q11.2DS [37].

There are no more reports on the changes in the pituitary gonadal axis in patients with 22q11.2DS associated with increasing age. After all, 22q11.2DS patients present heterogeneous presentations relevant to multi-organ dysfunction. The fertility and changes in the hypothalamo-pituitary–gonadal axis that happened in this case will also be used as a reference for future clinical management studies.

## Data Availability

All data that were generated or analyzed during the present study are contained in this article for publication.
